# Application of MALDI-TOF MS and FT-IR spectroscopy in identification and antibiotic resistance profiling of lactic acid bacteria

**DOI:** 10.1007/s00253-025-13550-x

**Published:** 2025-07-10

**Authors:** Tamás Kocsis, Anna Győrffy, Andrea Pomázi

**Affiliations:** Department of Food Microbiology, Hygiene, and Safety, Hungarian University of Agriculture and Life Sciences, 1118 Budapest, Hungary

**Keywords:** Antibiotic resistance, FT-IR Biotyper, Lactic acid bacteria, MALDI-TOF MS, Phenotypic profiling

## Abstract

**Abstract:**

This study evaluates the combined application of MALDI-TOF MS and Fourier transform infrared (FT-IR) spectroscopy for the purpose of species identification and strain-level differentiation of lactic acid bacteria isolated from commercial yoghurts. MALDI-TOF MS provided rapid species-level identification, while FT-IR detected metabolic variations by analysing key cellular components such as membrane fatty acids (3000–2800 cm^−1^), protein amide bands (1800–1500 cm^−1^), and polysaccharides (1200–900 cm^−1^). These spectral features reflected phenotypic differences among strains linked to antibiotic resistance profiles. Disc diffusion antibiotic susceptibility testing revealed significant variability among isolates, and a strong correlation was observed between FT-IR-derived linear discriminant analysis (LDA) clusters and resistance to oxacillin, clindamycin, and tetracycline (Chi^2^ test, *p* < 0.05). This demonstrates FT-IR as a rapid, non-invasive tool for the early detection of resistant strains, facilitating real-time monitoring of bacterial adaptation during fermentation. The findings of this study provide support for integrating FT-IR and MALDI-TOF MS into industrial quality control, aiding in strain selection and enhancing food safety.

**Key points:**

• *FT-IR enables rapid phenotypic typing of lactic acid bacteria strains.*

• *Spectral profiles correlate with antibiotic resistance.*

• *MALDI-TOF MS and FT-IR offer a robust, complementary identification method.*

## Introduction

Despite the widely recognised benefits of fermented dairy products, the emergence of antibiotic resistance in lactic acid bacteria (LAB) used in food production poses a growing public health concern (Kiskó et al. 2025). LAB are of significance not only for the sensory and nutritional quality of dairy products, but also as probiotics that benefit human health (Marco et al. 2017). In addition to milk, fermented dairy products or sour milk products have seen a marked increase in recent years due to their widely recognised beneficial physiological effects (Leroy & De Vuyst 2020). On the other hand, over the past decades, there has been a rise in allergic, gastrointestinal, and cancer-related diseases, possibly stemming from the improper functioning of the immune system (Koh et al. 2023). A significant challenge of the twenty-first century is the growing prevalence of antibiotic resistance, necessitating its effective address (Ventola 2015). A notable issue is that antibiotics, through their use, can infiltrate drinking water supplies, soil, and food, thereby contributing to the development of resistant strains (Berendonk et al. 2015). A number of recent studies have reported on the occurrence of antibiotic-resistant lactic acid bacteria (Temmerman et al 2003; Ammor et al 2007; Floris et al 2025). Therefore, it is imperative to note that only strains of lactic acid bacteria that have been verified to be devoid of acquired antibiotic resistance genes and possessing only natural, strain-specific “intrinsic” resistance can be marketed commercially as probiotic starter cultures, feed additives, or probiotics. This verification process is crucial in preventing the emergence of new resistant strains (Wong et al. 2015, Stefańska et al. 2021).

The extent to which LAB is used in fermented foods—particularly in commercially available yoghurts—harbours or transmits resistance traits remains insufficiently understood. This discrepancy in understanding is particularly disconcerting, given that probiotic microorganisms, like pathogens, can exhibit both intrinsic and acquired resistance. While intrinsic resistance is typically non-transferable and strain-specific, acquired resistance has been observed to disseminate via horizontal gene transfer mechanisms such as transformation, transduction, or conjugation (Mathur & Singh 2005; Ammor et al 2007; Gueimonde et al. 2013). Given these concerns, there is essential for the development of effective and reliable methods for identifying LAB strains and assessing their antibiotic resistance profiles. Modern microbiological diagnostics employ genotypic and phenotypic approaches. Genotypic methods analyse DNA-based traits that are stable under environmental influences, whereas phenotypic methods assess traits influenced by environmental factors (Madigan et al. 2018; Azrad et al. 2022). Recent advancements in the field have led to the emergence of techniques such as MALDI-TOF MS and FT-IR spectroscopy, which have been proven to be rapid and accurate phenotypic tools for bacterial identification and resistance profiling (Haider et al. 2025). Matrix-assisted laser desorption/ionisation–time of flight mass spectrometry has been identified as an alternative solution for identification (Lee et al. 2023). This analytical technique can analyse the mass-to-charge ratio of a multitude of biomolecules, including but not limited to peptides and proteins (Demirev & Fenselau 2008). In this proteomic approach, the proteins that are most readily identifiable include abundant and conserved ribosomal proteins, ribosomal modulating factors, cellular structural proteins, stored proteins, chaperones, and nucleic acid-binding proteins (Jadhav et al. 2014). It is possible to record and compare a characteristic fingerprint of organic matter with a reference list based on the mass spectrum information. This process can be used to identify the microbe at the species level. Fourier-transform infrared spectroscopy is a technique that analyses light absorption to gather information about the molecular composition of a sample (Azrad et al. 2022). The technique involves directing infrared radiation onto the prepared sample, thereby inducing molecular vibrations and facilitating the transition of molecules to higher energy states. These vibrations result in the absorption of light at specific wavelengths, creating a unique spectral pattern. The presence of nucleic acids, proteins, carbohydrates, and lipids within the sample has been shown to influence the spectral peaks observed in the FT-IR spectrum. FT-IR is a valuable method for identifying intraspecies differences in microorganisms (typing), with preparation and processing steps similar to those in MALDI-TOF analysis (Bisognin et al. 2022).

The present study was conceived to investigate the application of two analytical techniques—MALDI-TOF MS and Fourier-transform infrared (FT-IR) spectroscopy—for the identification of lactic acid bacteria isolated from commercial yoghurts, and to assess their antibiotic resistance profiles. The MALDI-TOF MS technique was selected due to its rapid, cost-effective, and accurate capabilities for microbial identification, making it particularly suitable for the routine analysis of large numbers of isolates. FT-IR was utilised as a complementary analytical method to assess phenotypic variation, with particular efficacy in discriminating functional traits such as potential antibiotic resistance. In comparison with conventional molecular or biochemical approaches, both techniques offer higher throughput, faster processing, and lower operational costs based on the phenotypic insights. The findings of this research may provide important insights into food safety assessment and contribute to public health efforts by addressing a salient concern: the extent to which lactic acid bacteria used in dairy production may harbour or transmit antibiotic resistance.

## Materials and methods

### Sample collection and storage

In this study, 18 commercially available yoghurt products were analysed, encompassing traditional cow’s milk yoghurts, goat’s milk yoghurts, and plant-based vegan alternatives from well-known manufacturers. The selection of vegan products included various plant-based fermented alternatives, such as coconut-, soy-, and almond-based yoghurts. In addition to plain yoghurts, flavoured yoghurt variants were also examined to provide variety. Flavoured yoghurts mainly contained added fruit and included variations such as sugar-free, low-fat, and lactose-free products. These variants were “light” (JBL) and sugar-free (JB0), as well as apple-cinnamon lactose-free Greek yoghurt (T) and layered blueberry yoghurt (N). Among plant-based alternatives, four commercially available products were selected to ensure diversity in raw materials. These included products were a coconut milk-based vegan yoghurt (ZK), two soy-based products—one plain (JS) and one fruit-flavoured (A)—and an almond-based yoghurt (MM).

Samples were obtained from local shops and supermarkets in March 2024. The samples were analysed immediately after their collection, or alternatively stored at 4 °C for a maximum 16 h prior processing. The products collected were labelled using the brand’s initial one or two letters. In certain cases, supplementary letters were utilised to indicate the diverse product variants. The characteristics of the products are summarised in Table 1.

**Table 1:** Commercially available yoghurts used in the measurements (*n* = 18)

*Abridgment*	*Raw material—milk*	*Type*	*Description*	*Exemption*	*The precise culture if indicated*	*Number of the manufacturer*
N	Cow	Fruity	Pasteurised milk, blueberry fruit preparation	None		1
JBL	Cow	Fruity	Fat content 1.1%, 9% strawberry, with sugar and sweetener, reduced energy content	Light (ow fat)		2
JB0	Cow	Fruity	Fat content 0.1%, 9% strawberry, no added sugar	No added sugar		2
MA	Cow	Natural	Fat content 3%	None		3
C	Cow	Natural	Fat content 4.5%	None		4
MILM	Cow	Natural	Fat content 3.6%, milk, milk protein conc., culture, lactase enzyme	Lactose-free		5
T	Cow	Fruity	Fat content 10%	Lactose-free	*Streptococcus thermophilus*	6
ZB	Cow	natural	Fat content 3.5%, organic	None		6
MI	Cow	natural	Fat content 3.6%	None		5
D	Cow	natural	Fat content 3.5%	None		7
Z	Cow	natural	Fat content 3.1%	None		2
G	Cow	Natural	Very dense	None	*Streptococcus thermophilus*, *Lactobacillus delbrueckii* subsp. *bulgaricus*, *L. acidophilus*, *Bifidobacterium lactis*	8
H	Cow	Natural	Fat content 2.8%, thick drinking yogurt	None		9
K	Goat	Natural		None		10
ZK	Plant-based	Natural	Creamy texture	Vegan, no added sugar		2
JS	Plant-based	Natural	Organic	Vegan, no added sugar		11
A	Plant-based	Fruity	Strawberries, cherries, dates, added Ca and vitamins, small fruit pieces, medium density	Vegan, no added sugar	*Streptococcus thermophilus*, *Lactobacillus bulgaricus*	12
MM	Plant-based	Fruity	Raspberry almond preparation, very dense with large pieces of fruit	Vegan		13

### Bacterial isolation and culture conditions

Yoghurt samples were homogenised and processed with diluent (1 g of peptone and 8.5 g of NaCl in 1000 mL of distilled water) in a 1:9 (w/v) ratio in Stomacher bags (Thermo Scientific, Oxoid™ AnaeroGen™ 2.5L Sachet). Serial tenfold dilutions were then performed, with the last three dilution levels being surface-plated on MRS agar (27.6 g of MRS broth powder (BioLab, BAA11000) and 7.5 g of agar in 500 mL of distilled water). Inoculated Petri dishes were then incubated under anaerobic conditions at 37 °C for 48 h (Lin et al. 2006). Separated distinct colonies (3–5 per plate) were then picked with sterile toothpicks and transferred into Eppendorf tubes with screw cap containing MRS broth. These were then incubated anaerobically (O_2_ < 0.1%, CO_2_ 7–15.0%), after which the cells were pelleted by centrifugation at 12,000 rpm for 10 min at 25 °C. The isolates obtained were directly used for experiments or maintained at − 80 °C. If multiple microbial isolates were obtained from the same product, they were numbered sequentially (e.g. N1, N2, N3).

The isolated strains are stored at − 80 °C in 10% glycerol at the Department of Food Microbiology, Hygiene and Safety, Hungarian University of Agriculture and Life Sciences, Budapest, Hungary.

### Identification by MALDI-TOF MS

The isolates were identified by implementing an extended direct transfer procedure, whereby each colony of isolates was meticulously placed onto a Bruker’s ground steel target plate. Subsequently, 1 µL of 70% formic acid was added, and following a period of air-drying, 1 µL of α-cyano-4-hydroxycinnamic acid matrix solution (HCCA) was incorporated (Hou et al. 2019). Notably, each bacterial sample was subjected to rigorous measurement on two separate occasions. The identification process was completed using MALDI Biotyper 4.1 (Bruker Daltonics GmbH & Co., Billerica, MA, USA). MALDI-TOF MS spectra of the samples were collected using a Microflex LT/SH (Bruker Daltonics GmbH & Co., Bremen, Germany) mass spectrometer equipped with a nitrogen laser (λ = 337 nm) at a laser frequency of 60 Hz (Fuchs et al. 2010). The instrument operated in linear positive ion detection mode under MALDI Biotyper 4.1 Realtime Classification (RTC) (Bruker Daltonics GmbH & Co., Bremen, Germany) and FlexControl 4.1 (Bruker Daltonics GmbH & Co., Bremen, Germany). Mass spectra were acquired in the 2000–21,000 Da range for each sample analysed for species-level microbial identification. MALDI-TOF MS spectra were generated from 240 single spectra, created in 40-laser-shot steps from random positions of each isolate. The system was calibrated before all measurements using *Escherichia coli* ribosomal protein standard (Bruker IVD Bacterial Test Standard, Bruker Daltonics GmbH & Co., Bremen, Germany) (Haider et al. 2023). FlexControl and FlexAnalysis (Bruker Daltonics GmbH & Co., Bremen, Germany) were utilised for the acquisition and processing of data. The FlexAnalysis software was then employed for the pre-processing of the mass spectra, a process that involved subtracting the baseline, applying a smoothing filter, and selecting peaks.

According to Bruker’s instructions, MALDI-TOF MS identification results were accepted at the genus or species level. A high-confidence identification is indicated by a score in the range of 2.00–3.00, which denotes reliable identification at the species level. Low-confidence identification is accepted at the genus level, with a score of 1.7–1.99. Scores below 1.7 are considered unreliable identifications at any level (Biswas & Rolain 2013).

### Typing by IR biotyper

In case of the FT-IR spectra acquisition and analysis, the bacterial samples were picked using an inoculation loop and transferred to 50 µL of 70% (vol/vol) ethanol in 1.5 mL tubes containing sterile metal rods for improved homogenisation (Bruker Daltonik GmbH & Co., Bremen, Germany). After thorough vortexing, 50 µL of sterile H_2_O was added. Then, 15 µL of the resulting bacterial suspension was added to a silicon sample plate (Bruker Daltonik GmbH & Co., Bremen, Germany) and dried at 37 °C for approximately 20 min (Cordovana et al. 2022). Four technical replicates of all isolates were analysed in each of three independent experiments on a commercially available IR Biotyper system (Bruker Daltonik GmbH & Co., Bremen, Germany) using the IR Biotyper software (version 4.0) with default analysis settings (32 scans per technical replicate; spectral resolution, 6 cm^−1^; apodisation function, Blackman-Harris 3-term; zero filling factor, 4). Measurements that did not meet the default quality criteria (0.4 < absorbance < 2; signal-to-noise ratio, < 40; fringes [× 10^−6^], < 100) were excluded from further analysis. The FT-IR instrument was calibrated before each measurement using a polystyrene standards (IRTS 1,2, Bruker Daltonics GmbH & Co., Bremen, Germany). The spectral processing is based on the second derivative of the 1300 to 800 cm^−1^ wavenumber range of the spectra, as recommended by the manufacturer. This processing was conducted using OPUS (version 9.0). After vector normalisation, the respective summary spectra were calculated as the average of the underlying (technical or biological) replicate spectra. The similarity between all isolate spectra was calculated to generate a matrix, which was then used to cluster the isolates using the average linkage algorithm of the IR Biotyper software. Finally, linear discriminant analysis (LDA) was applied to the obtained results, and the findings were visualised as clusters in the “Results” section.

### Antibiotic sensitivity testing of isolates

Lactic acid bacteria were grown in 48-h cultures on MRS plates and used to prepare bacterial suspensions with densities 0.4 McFarland units. The antibiotic sensitivity testing was performed using the Kirby-Bauer method (Kirby & Bauer 1966) and the standard guidelines (CLSI 2015a, 2015b, 2015c; EUCAST 2025), with minor modifications. A microbial lawn was prepared on MRS agar plates and antibiotic-impregnated discs were placed upon it. The plates were then incubated under anaerobic conditions at 37 °C for 48 h, after which the diameters of the inhibition zones formed around the antibiotic discs were measured. The experiments were performed in three parallel. For interpretation of the results, the recommendation of CLSI M45 (2015b) and EFSA (2018) documents was followed. The antibiotics evaluated in this study encompassed penicillin (10 µg), oxacillin (5 µg), clindamycin (10 µg), and tetracycline (30 µg). The selection of these antibiotics was predicated on their divergent mechanisms of action. Oxacillin and penicillin function as cell wall synthesis inhibitors, while clindamycin and tetracycline act as protein synthesis inhibitors. The evaluation criteria used in this study are summarised in Table 2.

**Table 2:** Interpretation criteria of inhibition zone diameters for antibiotics used in this study

Antibiotics	Mechanism of action	Zone of inhibition^a^ (mm)			References
		R	I	S	
Oxacillin (5 µg)	Inhibition of cell wall synthesis	≤ 14	15–19	≥ 20	Khan, et al. (2019)
Penicillin (10 µg)		≤ 14	-	≥ 15	CLSI M100 (2015c)
Clindamycin (10 µg)	Inhibition of protein synthesis	≤ 8	9–11	≥ 12	Charteris et al. (1998)
Tetracycline (30 µg)		≤ 14 ≥	15–18	19	CLSI M100 (2015c), Charteris et al. (1998)

^a^Diameter of inhibition zone to classify bacteria as resistant (R), intermediate (I) or sensitive (S)

### Correlation analysis

To investigate the correlation between FT-IR data and antibiotic susceptibility profiles of microbial isolates, several statistical tests were conducted. The isolates were initially classified according to the results of linear discriminant analysis (LDA) applied to the FT-IR spectral data. Specifically, LDA scores were divided into quartiles, and isolates were grouped into four categories (groups 1 to 4) according to their position within these quartiles. Subsequently, an examination was conducted into the correlation between the aforementioned FT-IR-based groups and the manifestation of antibiotic resistance phenotypes. This was undertaken utilising Chi-square (χ^2^) tests for independence. The Chi-squared test was selected to determine whether there is a statistically significant relationship between the categorical variables of FT-IR LDA-derived groups and antibiotic susceptibility categories (sensitive/resistant). In light of the presence of cells with minimal expected counts, Fisher’s exact test was also implemented to provide an exact significance value. This method is more reliable in instances of sparse data. Monte Carlo simulations (based on 10,000 replicates) were also employed to estimate *p*-values for both Chi-square and Fisher’s exact tests, thus offering robust significance estimates when asymptotic assumptions might be violated. The magnitude of the association between variables was quantified by calculating Cramér’s V, which provides a metric of effect size for nominal data. Values approaching 1 indicate a stronger association. All statistical analyses were conducted using IBM SPSS Statistics software. The significance of these results was determined at *p* < 0.05.

## Results

### Isolation of lactic acid bacteria

Lactic acid bacteria were successfully isolated from plain yoghurts (MI, Z), lactose-free yoghurt (MILM), bio yoghurt (ZB), Greek yoghurt (G), and goat’s milk yoghurt (K), as well as apple-cinnamon lactose-free Greek yoghurt (T) and layered blueberry yoghurt (N). In case of plant-based fermented alternatives, lactic acid bacteria were isolated from coconut milk-based vegan yoghurt (ZK), the soy-based plain yoghurt (JS), and almond-based yoghurt (MM). A total of 40 LAB isolates were obtained during the isolation process. However, it should be noted that lactic acid bacteria were not be recovered from six fermented products (JBL, JB0, MA, C, D, A).

### Identification by MALDI-TOF MS

Starter cultures are typically comprised of a mixture of multiple species. Based on the results obtained from MALDI-TOF analysis, *Streptococcus salivarius* ssp. *thermophilus*, *Lactobacillu*s *delbrueckii*, *Lacticaseibacillus rhamnosus*, *Lactiplantibacillus plantarum*, and *Lactobacillus acidophilus* were successfully isolated and identified (Table 3). According to the results obtained, at the time of the analysis, certain products contained only a single predominant lactic acid bacterium species in high numbers. In the T, ZB, and ZK samples, *Streptococcus salivarius* ssp. *thermophilus* was detected in significant quantities. In contrast, multiple species were successfully identified in the MI, MILM, and G samples. The probiotic effects of live-culture products have been well-documented in the literature (Marco et al. 2017).

**Table 3:**
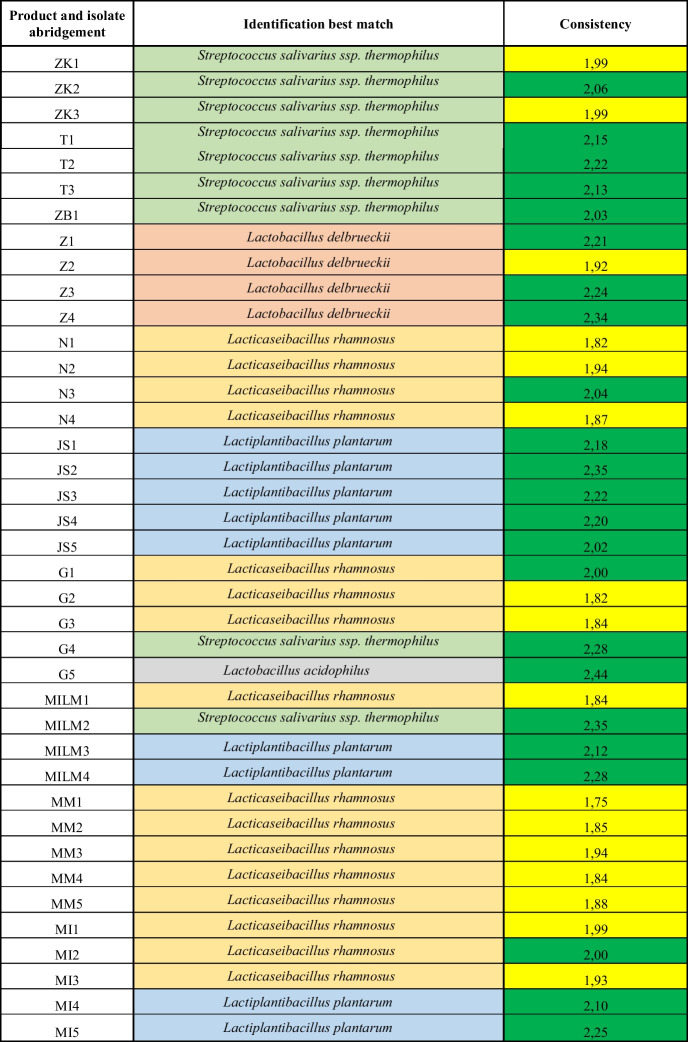
Results of the identification of lactic acid bacteria isolates using MALDI-TOF MS. Reliability scores: high (2.00–3.00), isolates identified at the species level; low (1.77–1.99), isolates identified at the genus level

A comparison of dairy-based and plant-based products revealed no significant differences in the occurrence of the isolated and identified species. In one vegan product (JS), *Lactiplantibacillus plantarum* was identified, irrespective of the fruit-flavoured or plain nature of the product. In the MILM product, this species was detected alongside *Lacticaseibacillus rhamnosus* and *Streptococcus salivarius* ssp. *thermophilus*, despite being primarily used in the fermentation of plant-based products due to its enhanced growth in plant-derived media. However, it also thrives in dairy-based fermented products (Horáčková et al. 2022).

### FT-IR typing and LDA analysis

**Fig. 1:**
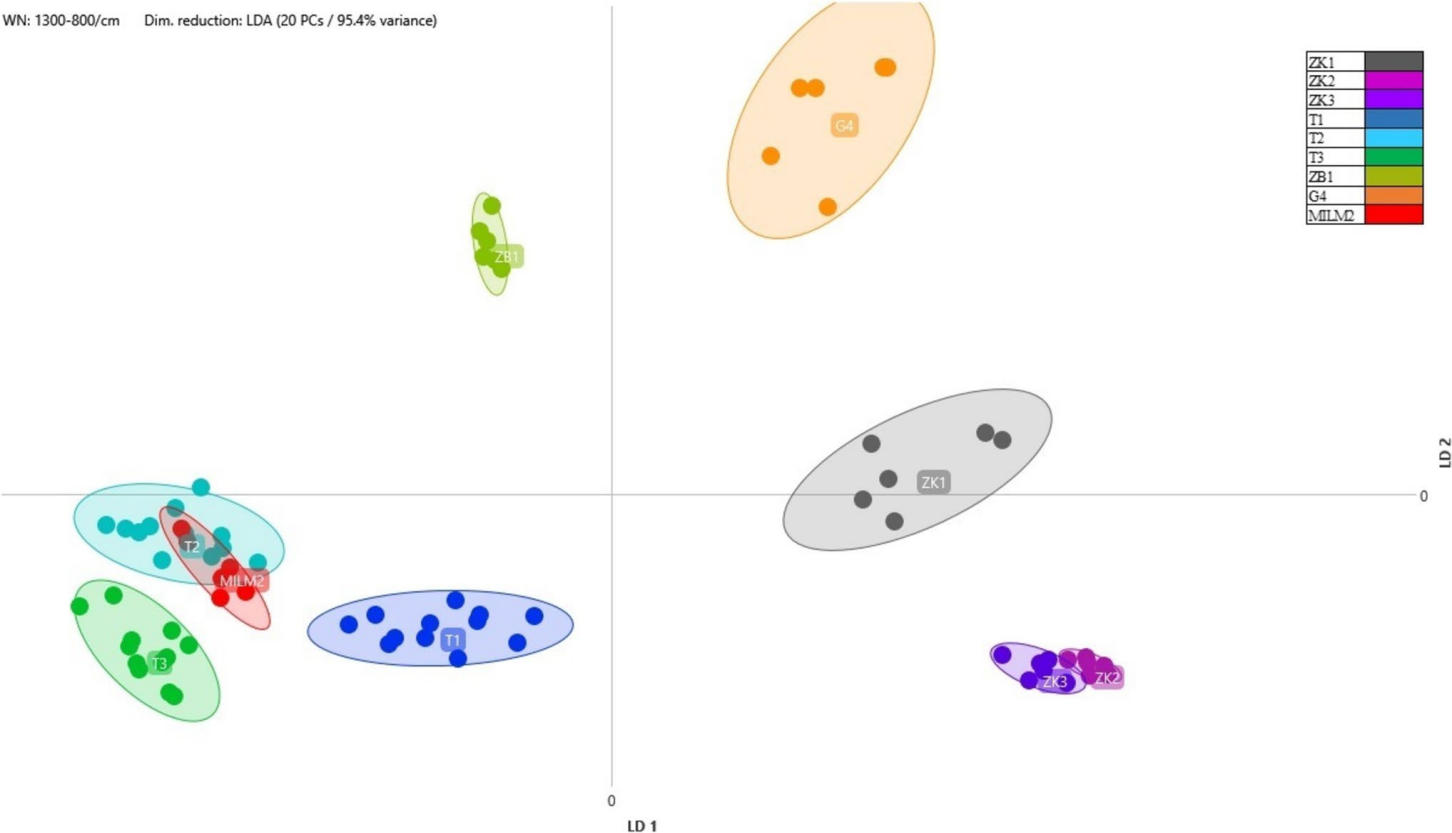
Comparison of *Streptococcus salivarius* ssp. *thermophilus* strains isolated from different samples using LDA at a 95.4% variance level. The colour coding of the individual isolates is as follows: ZK1, grey; ZK2, violet; ZK3, purple; T1, dark blue; T2, light blue; T3, dark green; ZB1, light green; G4, orange; MILM2, red

**Fig. 2:**
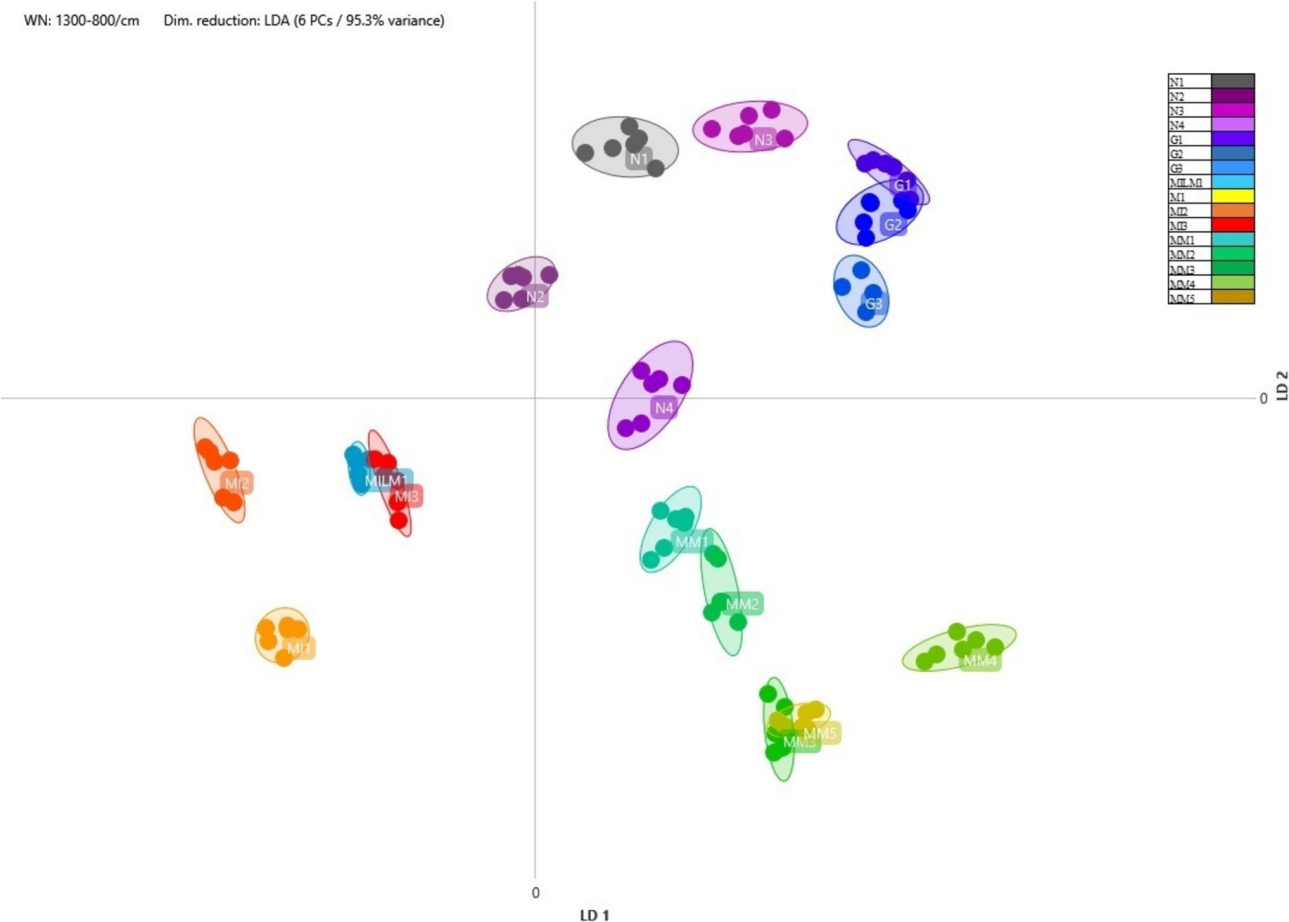
Comparison of *Lacticaseibacillus rhamnosus* strains isolated from different samples using LDA at a 95.3% variance level. The colour coding of the individual isolates is as follows: N1, grey; N2, purple; N3, purple; N4, violet; G1–G3, shades of blue; MILM1, shades of blue; M1, yellow; MI2, orange; MI3, red; MM1–MM4, shades of green; MM5, brown

**Fig. 3:**
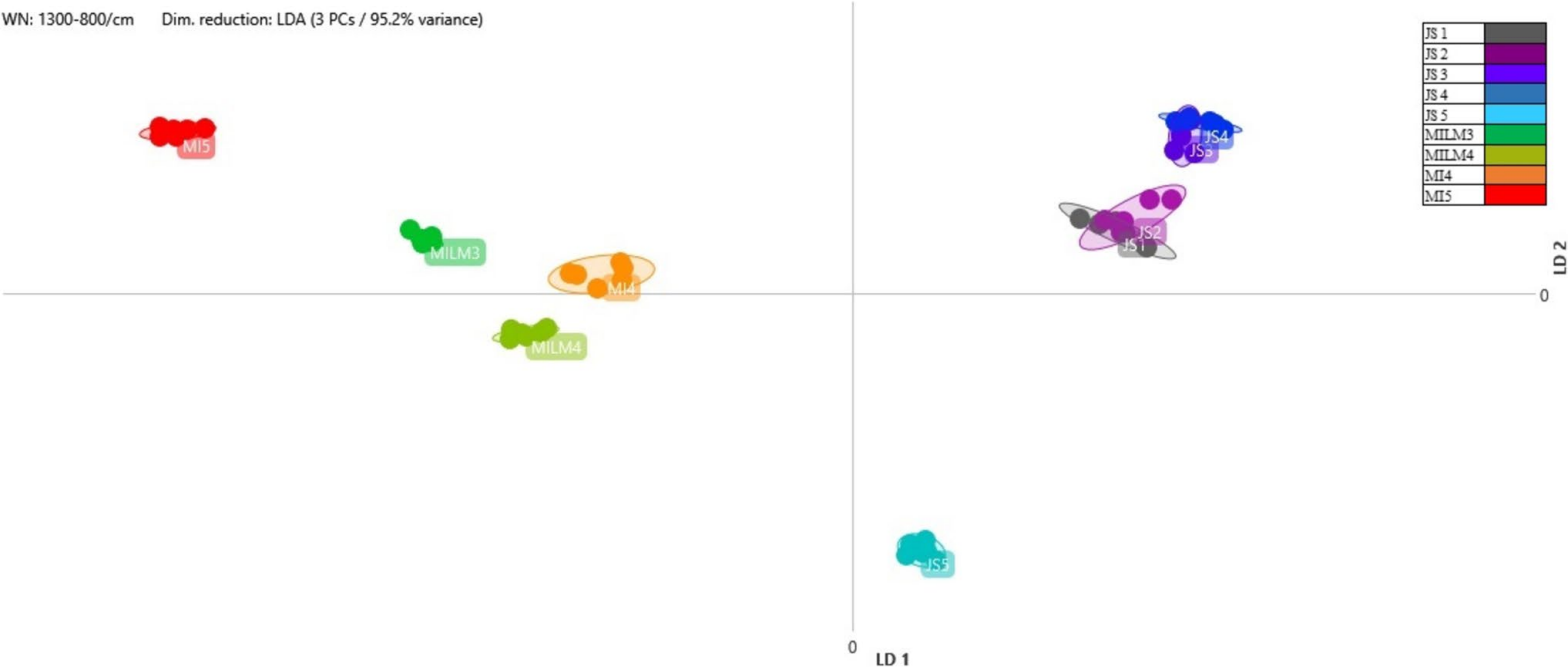
Comparison of *Lactiplantibacillus plantarum* strains isolated from different samples using LDA at a 95.2% variance level. The colour coding of the individual isolates is as follows: JS1, grey; JS2, purple; JS3, bluish purple; JS4, dark blue; JS5, light blue; MILM3, green; MILM4, yellow-green; MI4, orange; MI5, red

The values obtained from the analysis of FT-IR absorbance data using linear discriminant analysis (LDA) are presented in Figs. 1, 2, and 3. The differentiation of the isolates previously identified at the species level by MALDI-TOF was successfully achieved based on the distances between clusters represented in the two-dimensional coordinate system, according to their carbohydrate composition.

A total of nine strains of *Streptococcus salivarius* ssp. *thermophilus* were isolated, designated as ZK1, ZK2, ZK3, T1, T2, T3, ZB1, G4, and MILM2 (Table 3). The outcomes of the linear discriminant analysis performed on the *Streptococcus salivarius* strains’ absorbance data, isolated from five distinct yoghurt samples using Fourier-transform infrared spectroscopy, are presented in Fig. 1.

The proximity of two isolates in the plot indicates the similarity of their metabolic profiles. For each isolate, an occurrence zone was delineated around the data points following a minimum of three independent sample preparations and measurements. In instances where the zones overlap, it can be deduced that the isolates belong to the same strain, as evidenced by the overlap observed in ZK2 and ZK3, or MILM2 and T2. Conversely, where zones do not overlap, there is a significant difference in metabolic products (e.g. T1, T2, and T3). The greater the distance between clusters, the more pronounced these differences are (e.g. G4 and T1).

Sixteen strains of the *Lacticaseibacillus rhamnosus* species were successfully isolated: N1, N2, N3, N4, G1, G2, G3, MILM1, MI1, MI2, MI3, MM1, MM2, MM3, MM4, and MM5 (see Table 3 for details). The linear discriminant analysis (LDA) results demonstrated that the zones of multiple isolates from different samples overlapped, indicating close similarity. For instance, overlaps were observed between MM3 and MM5, G1 and G2, and MILM1 and MI3 (Fig. 2). Given that the clustering results primarily correlate with the product groups of different manufacturers, it is likely that the producers use the same starter cultures for fermenting their respective products. This finding is consistent with the conclusions of earlier studies, which indicate that the production of industrial yoghurt and probiotic dairy products frequently relies on standardised microbial consortia. The rationale behind this is to ensure consistent fermentation performance and product quality.

Nine strains of the *Lactiplantibacillus plantarum* species were detected. Based on the linear discriminant analysis (LDA) results, the isolates were classified into three main clusters, of which a high degree of similarity was observed among the JS1, JS2, JS3, and JS4 samples, while JS5 exhibited distinct characteristics. The third cluster consisted of the MILM3, MILM4, MI4, and MI5 isolates (Fig. 3). The results suggest that specific *L. plantarum* strains are consistently present in certain product groups, possibly reflecting standardised fermentation practices by manufacturers. The distinct separation of JS5 may indicate strain variation due to differences in fermentation conditions, raw material composition, or additional microbial interactions. Further analyses could provide deeper insights into the functional differences between these clusters and their potential impact on product characteristics, such as probiotic functionality, flavour development, and metabolic activity.

### Antibiotic susceptibility testing

Antibiotic susceptibility testing was performed on 15 isolates in three replicates, selected to represent the identified species and, where possible, derived from different products. The results of the inhibition zone test are presented in Table 4. For oxacillin, isolates belonging to *Lactiplantibacillus plantarum*, *Lactobacillus delbrueckii*, and *Streptococcus salivarius* ssp. *thermophilus* exhibited similar sensitivity, with inhibition zone diameters ranging from 20 to 23 mm. However, sensitivity differences were observed among *Lacticaseibacillus rhamnosus* strains, with the N4 sample being resistant to oxacillin, which correlates with the FT-IR analysis shown in Fig. 2, where it appears as an outlier, distinct from other clusters. The inhibition zones for the MI2 and MILM1 samples were considered to demonstrate transitional sensitivity. Based on the LDA (linear discriminant analysis) figure, they were grouped separately from the other oxacillin-sensitive strains, such as G2, G3, MM4, and MM5. The G5 sample, representing *Lactobacillus acidophilus*, demonstrated the highest sensitivity to this antibiotic.

**Table 4:**
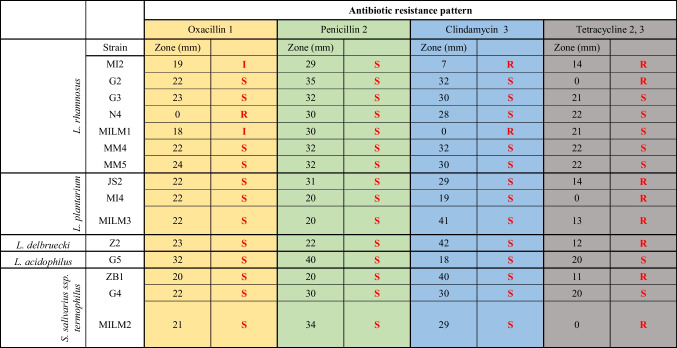
Results of disc diffusion susceptibility tests (Kirby–Bauer) with lactic acid bacteria strains (*n* = 45), after 48 h. Notations: *S* sensitive, *I* intermediate, *R* resistant

1: Khan et al. (2019). Presence of blaCTX-M antibiotic resistance gene in *Lactobacillus* spp. isolated from Hirschsprung diseased infants with stoma. Journal of infection in developing countries, 13(5), 426–433. https://doi.org/10.3855/jidc.10968

2: CLSI. M100-S25 (2015c) Performance standards for antimicrobial susceptibility testing; Twenty-fifth informational supplement, Wayne, PA: Clinical and Laboratory Standards Institute

3: Charteris et al. (1998). Antibiotic susceptibility of potentially probiotic *Lactobacillus* species. Journal of Food Protection 61(12):1636–43. https://doi.org/10.4315/0362-028X-61.12.1636

All the strains tested exhibited sensitivity to penicillin, and by the oxacillin tests, the G5 *Lactobacillus acidophilus* isolate demonstrated the highest level of sensitivity. Based on the results for both antibiotics, it can be concluded that *Lactobacillus acidophilus* may be particularly sensitive to cell wall synthesis inhibitors; however, further investigations are required to confirm this statement. When testing clindamycin, variable results were obtained for the lactic acid isolates. For *Lacticaseibacillus rhamnosus*, the MILM1 and MI2 isolates demonstrated reduced sensitivity and resistance compared to the other samples, including G2, G3, N4, MM4, and MM5 isolates. *Streptococcus salivarius* ssp. *thermophilus* strains exhibited sensitivity to clindamycin, albeit to varying extents, with the ZB1 isolate demonstrating greater sensitivity than the others. In the case of *Lactiplantibacillus plantarum*, the antibiotic induced inhibition zones of varying sizes across three isolates. Of particular interest is the observation that the MILM3 (highly sensitive) and MI4 (transitionally sensitive) isolates were closely related according to Fig. 3, yet they exhibited significant differences in sensitivity to clindamycin. The inhibition zones induced by tetracycline also demonstrated variability. Based on the response to tetracycline, the microbes could be divided into two groups: sensitive or resistant, with no strains showing transitional inhibition zone sizes. The resistant strains (MI2, G2, JS2, MI4, MILM3, Z2, ZB1, MILM2) and sensitive strains (G3, N4, MILM1, MM4, MM5, G5, G4) were distributed approximately evenly. The G2 and G3 strains showed different sensitivity to Tetracycline.

### Association between FT-IR spectral clusters and antibiotic susceptibility

The results of the relationship between FT-IR spectroscopy and antibiotic susceptibility profiles are shown in Table 5. Statistically significant associations were observed between FT-IR spectral groups and resistance patterns for three antibiotics tested (oxacillin, clindamycin, and tetracycline; *p* < 0.001). Cramér’s V values ranged from 0.627 to 0.819, indicating strong associations. These findings suggest that strains with similar FT-IR profiles tend to exhibit similar antibiotic resistance characteristics. These findings indicate that strains with similar FT-IR profiles tend to exhibit similar antibiotic resistance characteristics. All antibiotics were tested at concentrations recommended by clinical diagnostic guidelines. Since all isolates were susceptible to penicillin at the applied dose, the Chi-square test could not be performed for this antibiotic. The contingency table contained cells with zero observed and/or expected frequencies across categories, thus violating the test’s hypothesis.

**Table 5:** Statistical associations between FT-IR spectral clusters and antibiotic sensitivity, based on the Chi-square test, Fisher’s exact test, and Monte Carlo simulation at a 95% confidence interval

Variable pair	Chi^2^ value	df	Asymp. p	Fisher’s exact p	Monte Carlo p	Cramér’s V	Significance (*p* < 0.05)
FT-IR × oxacillin	55.032	6	< 0.001	< 0.001	< 0.001	0.627	Yes
FT-IR × clindamycin	46.974	3	< 0.001	< 0.001	< 0.001	0.819	Yes
FT-IR × tetracycline	30.751	3	< 0.001	< 0.001	< 0.001	0.663	Yes

## Discussion

### Species differentiation and consistency

Based on the MALDI-TOF identification results, the classification of isolates primarily corresponds to the manufacturers’ product groups, suggesting that producers frequently rely on identical or highly similar starter cultures to ferment the products tested. This finding aligns with the results of Murphy et al. (2021). The results of FT-IR analysis, combined with linear discriminant analysis (LDA), provided valuable insights into the metabolic profiles of different bacterial strains isolated from yoghurt samples. The successful species-level differentiation of isolates previously carried out by MALDI-TOF demonstrated the utility of FT-IR as a reliable method for distinguishing bacterial strains based on their carbohydrate composition. This approach was particularly effective in categorising *Streptococcus salivarius* ssp. *thermophilus*, *Lacticaseibacillus rhamnosus*, and *Lactiplantibacillus plantarum* strains which are commonly used in the production of yoghurt and other fermented dairy products. The proximity of isolates in the LDA plots suggested that strains with similar metabolic profiles clustered together, supporting the hypothesis that strains from the same product or production environment tend to have closer biochemical similarities. For example, the overlap between isolates such as ZK2 and ZK3 or MILM2 and T2 indicated that these isolates were likely to be the same strain. In the case of *Streptococcus salivarius* ssp. *thermophilus*, isolates from different yoghurt samples were found to have various degrees of metabolic differentiation. The clear separation of isolates such as T1, T2, and T3 from others, such as G4, suggests that fermentation conditions, raw material composition, or microbial interactions may play a significant role in shaping the final metabolic profile of the strains. These results highlight the importance of environmental factors in the fermentation process and their potential impact on the consistency and quality of dairy products. Similarly, the analysis of *Lacticaseibacillus rhamnosus* isolates showed overlapping zones in the LDA plots, indicating a high degree of similarity between strains from different producers. This overlap suggests that standardised fermentation practices, often involving the use of similar or identical starter cultures, may contribute to the homogeneity of bacterial strains used in industrial yoghurt production. The results for *Lactiplantibacillus plantarum* isolates showed a more pronounced differentiation, with apparent clustering of strains such as JS1, JS2, JS3, and JS4, while JS5 was separated. The functional implications of these differences could be significant, particularly in terms of probiotic activity, flavour development, and metabolic properties. Further investigation of these clusters could reveal important details about the potential health benefits and quality attributes of fermented products, as well as the role of specific strains in influencing product consistency and consumer acceptance. Beyond phenotypic clustering, these observations prompted further investigation into the functional traits of the isolates, particularly their antibiotic susceptibility, to explore potential correlations between metabolic profiles and resistance mechanisms.

### Insights into antibiotic resistance

To complement the phenotypic characterisation, antimicrobial susceptibility profiles were evaluated, aiming to assess whether differences in resistance align with the strain-level differentiation observed through FT-IR and LDA analyses. Regarding antibiotic susceptibility testing, it is important to note that there are currently no established thresholds for lactic acid bacteria; therefore, the results were evaluated according to the different guidelines and publications. In conclusion, the tested lactic acid bacterial strains exhibited more consistent susceptibility patterns to cell wall synthesis inhibitors, such as oxacillin and penicillin. In contrast, protein synthesis inhibitors, particularly tetracycline, revealed greater variability, with a higher proportion of resistant isolates observed in the case of multiple species as well. The presence of tetracycline resistance genes, including *tet* K, M, O, Q, S, W, and 36, has been documented in various species of lactic acid bacteria (Ammor et al 2007). In recent years, studies have suggested a possible link between phenotypic characteristics determined by FT-IR strain typing and the antibiotic resistance profiles of bacterial strains. Studies have shown that metabolic fingerprints obtained by FT-IR spectroscopy can reflect certain traits associated with bacterial resistance mechanisms, particularly those related to cell wall synthesis and membrane structure (Barrera-Patiño et al. 2023). For example, FT-IR has been used to distinguish between antibiotic-resistant and sensitive strains based on subtle differences in their biochemical profiles, such as the presence of specific exopolysaccharides or cell wall modifications. International studies have investigated the feasibility of using FT-IR spectroscopy to identify antibiotic resistance in lactic acid bacteria and other pathogens. A study by Li et al. (2022) demonstrated that FT-IR can reliably differentiate between resistant and sensitive strains of *Lactobacillus* species, correlating specific spectral peaks with resistance to standard antibiotics such as ampicillin and tetracycline.

In the case of oxacillin, isolates belonging to *Lactiplantibacillus plantarum*, *Lactobacillus delbrueckii*, and *Streptococcus salivarius* ssp. *thermophilus* species showed similar susceptibility, with inhibition zones ranging from 20 to 23 mm. This consistent result across these species suggests that these strains share standard mechanisms of resistance or sensitivity to cell wall synthesis inhibitors, possibly due to structural similarities in their cell walls. However, a notable deviation was observed in the *Lacticaseibacillus rhamnosus* strains, where sample N4 was resistant to oxacillin. The resistance of strain N4 to oxacillin is significant as it was identified as an outlier in the FT-IR analysis, clustering separately from the other strains. This finding may indicate the presence of unique resistance mechanisms in this strain, possibly related to altered cell wall composition. In addition, the reduced susceptibility observed in the MI2 and MILM1 isolates, which were grouped separately in the LDA plot, indicates a gradation of resistance, suggesting that these strains may have developed partial resistance, which could be indicative of adaptive mechanisms or intermediate resistance.

Following the variable results obtained for oxacillin, particularly among *Lacticaseibacillus rhamnosus* strains, a more uniform susceptibility pattern was observed in the case of penicillin. The *Lactobacillus acidophilus* isolate (G5) showed the highest sensitivity. This result is consistent with the known effectiveness of penicillin against many Gram-positive bacteria, which typically have cell walls that are susceptible to this antibiotic. As *Lactobacillus acidophilus* showed the highest sensitivity to both oxacillin and penicillin, it is plausible that this species may be particularly sensitive to cell wall synthesis inhibitors.

Clindamycin testing revealed a more complex pattern of resistance, with variable results between strains. *Lacticaseibacillus rhamnosus* isolates, particularly MILM1 and MI2, exhibited reduced susceptibility and even resistance compared to other strains, such as G2, G3, and MM4. This variability suggests that *Lacticaseibacillus rhamnosus* strains may employ different mechanisms of resistance to protein synthesis inhibitors, such as modifying ribosomal targets or possessing efflux systems that reduce antibiotic accumulation (Rodriguez et al. 2024). In contrast, *Streptococcus salivarius* ssp*. thermophilus* strains showed consistent susceptibility to clindamycin, although the ZB1 isolate showed a higher degree of susceptibility, which may be due to intrinsic factors such as the presence of specific efflux pumps or membrane permeability (Stašková et al. 2021). In the context of *Lactiplantibacillus plantarum* isolates, clindamycin susceptibility demonstrated significant variability. Specifically, MILM3 exhibited high susceptibility, while MI4 displayed sensitivity. The clustering of MILM3 and MI4 in the LDA analysis, despite their different responses to clindamycin, suggests that FT-IR fingerprinting, while effective in capturing metabolic phenotypes, may not reliably distinguish functional traits such as antibiotic resistance, which are often governed by genetic elements unrelated to primary metabolism. The marked difference in susceptibility between these two isolates highlights the complex nature of antibiotic resistance, which does not necessarily correlate directly with phenotypic clustering based on metabolic fingerprints, particularly in the context of FT-IR spectra.

The results for tetracycline demonstrated a clear distinction between sensitive strains and those that were resistant, with no intermediate sensitivity observed. The division of strains into two groups—resistant and sensitive—suggests that resistance to tetracycline in these isolates may be associated with specific resistance genes or efflux mechanisms that are either fully present or absent, without a gradation of sensitivity. The differential susceptibility observed in strains such as G2 and G3 further supports the idea that genetic variability plays a key role in determining antibiotic resistance profiles. Resistance in strains such as MI2, G2, and JS2 may indicate the presence of specific resistance determinants in the cell wall. In contrast, the sensitive strains, such as G3, N4, and G5, may lack these components.

### Implications for industry and future research

It is significant to observe that six isolates (JBL, JB0, MA, C, D, A) could not be identified using the applied MALDI-TOF MS. This may be attributable to the constraints imposed by the reference database, as certain artisanal or environmental strains commonly encountered in fermented products may not have been incorporated into standard spectral libraries. Consequently, the inability to classify these isolates highlights the microbial diversity present in commercially available yoghurt products. It suggests that some strains may represent either rare, underrepresented species or potential novel taxa. This observation supports the need for the implementation of complementary molecular identification methods (e.g. 16S rRNA, whole-genome sequencing) in future studies, thereby facilitating a more comprehensive understanding of their taxonomy and potential functional roles (Kahraman-Ilıkkan 2024). From the perspectives of food safety and probiotic efficacy, the presence of unidentified strains underscores the significance of comprehensive characterisation, as these microbes may carry unique metabolic or antibiotic resistance traits that were not captured in the present analysis. The observed metabolic diversity among lactic acid bacterial strains, as revealed by FT-IR spectroscopy, carries meaningful implications for the fermented food industry. Variations in carbohydrate and protein metabolism have been demonstrated to influence organoleptic properties, including flavour, aroma, and texture (Forde and Bolhuis 2022). These properties are critical parameters in consumer acceptance. It is acknowledged that strains exhibiting distinct metabolic profiles can result in divergent concentrations of organic acids, exopolysaccharides, or volatile compounds (Silva et al. 2023). This, in turn, has the potential to influence both the shelf life and the taste of the final product. Furthermore, specific metabolic traits may modulate probiotic efficacy by influencing colonisation potential or interaction with host microbiota (Bubnov et al. 2018). The recognition and characterisation of such phenotypic differences through the utilisation of rapid spectroscopic techniques has the potential to inform the development of tailored starter cultures that optimise both technological performance and health benefits.

The observed strong correlations between FT-IR spectral clusters and antibiotic resistance profiles suggest that FT-IR spectroscopy has potential as a rapid phenotypic screening tool for monitoring microbial populations in industrial fermentation processes. This could assist in the early detection of resistant strains and improve quality control measures, particularly in standardised starter cultures used for yoghurt production. Consistent findings were reported by Ilgaz and Kadiroglu (2022), who found that cholesterol modulates antibiotic resistance in *Salmonella typhimurium*, increasing the minimum inhibitory concentration (MIC) values for several antibiotics and altering cell structure, as detected by FT-IR spectroscopy. These findings underscore the global challenge of antimicrobial resistance and illustrate how environmental factors, such as cholesterol, can impact bacterial susceptibility. A significant disparity exists between the fermentation processes of artisanal and industrial origin. Marked differences in microbial exposure to stressors, such as salt concentration, pH, temperature, and competitive microbial interactions, characterise this disparity (Benedek et al. 2022). These stressors can modulate resistance phenotypes. It is evident that specific resistance mechanisms, including the upregulation of efflux pump expression and changes in membrane permeability, are known to occur under sublethal stress conditions, which are prevalent in food processing environments (Jánosity et al. 2023). Furthermore, the potential for the persistence of mobile genetic elements within these communities cannot be discounted; thus, additional investigation through genomic analysis is necessary. Taken together, these findings suggest that future research should aim to integrate genotypic and phenotypic data with production metadata to understand better the adaptive and potentially transferable nature of resistance traits in food-associated microbial populations.

## Conclusion

This study definitively demonstrates that combining MALDI-TOF MS and FT-IR spectroscopy provides a robust and rapid approach for identifying and characterising lactic acid bacteria in fermented dairy products. A key finding is the statistically significant correlation between FT-IR-derived LDA clusters and antibiotic resistance profiles for oxacillin, clindamycin, and tetracycline (Chi^2^ test, *p* < 0.05). This validates FT-IR as a reliable, non-invasive method for rapid resistance profiling, representing a novel application that fills a gap in current phenotypic screening methodologies. The present study makes a significant contribution to the existing literature by providing empirical evidence that strain-level variation in probiotic bacteria directly affects resistance patterns. This insight enables manufacturers to make informed choices in selecting starter cultures that promote health benefits while minimising antimicrobial resistance risks, thus advancing food safety and public health purposes. The study also highlights the limitations of MALDI-TOF MS in identifying artisanal or underrepresented strains, underscoring the need to complement spectroscopic methods with gene sequencing for complete taxonomic resolution. The integration of FT-IR into standard industrial workflows enables producers to enhance quality control, ensure batch consistency, and comply with mounting regulatory requirements for probiotic strain transparency and safety. These findings represent a significant milestone in innovation for the field of analytical microbiology, offering practical strategies for monitoring antimicrobial resistance within the food chain. Subsequent studies should investigate the genetic basis of resistance in these strains, as well as examine the influence of their metabolic profiles on the development of resistance.

## Data Availability

Data will be made available on request.
